# The role of SIRT1 in BMP2-induced chondrogenic differentiation and cartilage maintenance under oxidative stress

**DOI:** 10.18632/aging.103161

**Published:** 2020-05-22

**Authors:** Yang Lu, Li Zhou, Lijun Wang, Shan He, Honglei Ren, Nian Zhou, Zhenming Hu

**Affiliations:** 1 Department of Orthopaedic Surgery, The First Affiliated Hospital of Chongqing Medical University, Yuzhong, Chongqing 400016, China; 2 Department of Oncology, The First Affiliated Hospital of Chongqing Medical University, Chongqing 400016, China; 3 Department of Orthopaedic Surgery, The People's Hospital of Dazu, Chongqing 402360, China

**Keywords:** cartilage defect repairing, MSCs, chondrogenic differentiation, bone morphogenetic protein 2 (BMP2), silent mating type information regulator 2 homolog-1 (SIRT1)

## Abstract

Articular cartilage defects are common in the clinic but difficult to treat. Exploring the chondrogenic molecular mechanisms of mesenchymal stem cells (MSCs) is of great theoretical interest and industrial significance. Bone morphogenetic protein 2 (BMP2) is a key factor that induces cartilage differentiation and can induce stem cell chondrogenic differentiation. However, the oxidative stress in the microenvironment during cartilage injury and degeneration inhibits cartilage regeneration and homeostasis. Silent mating type information regulator 2 homolog-1 (SIRT1) is an important histone deacetylase that regulates proliferation, differentiation, aging, and inflammation processes; moreover, it is an essential factor for chondrogenesis. The specific mechanism of SIRT1 in cartilage differentiation and homeostasis is still unclear. First, we investigated whether SIRT1 could coordinate BMP2-induced chondrogenic differentiation. Second, we investigated the protective effect of SIRT1 on BMP2-induced MSCs under oxidative stress. The results showed that SIRT1 could promote BMP2-induced chondrogenic differentiation of MSCs, and reduce the apoptosis and decomposition of extracellular matrix under oxidative stress. In summary, these results suggested that SIRT1 plays an important coordination role in BMP2-induced chondrogenic differentiation of stem cells and cartilage maintenance under oxidative stress, establishing the experimental basis for exploring the use of SIRT1 in cartilage defect repair.

## INTRODUCTION

There are many causes of cartilage injury, such as inflammation, aging, and oxidative stress [1, 2]. However, articular cartilage is a highly differentiated tissue that lacks a blood supply, lymph, and nerves, resulting in poor self-healing ability [3–5]. Currently, the main methods for treating cartilage defects are merely focused on relieving pain symptoms and delaying degeneration, and it is difficult to achieve successful healing [6]. Mesenchymal stem cells (MSCs), one type of mesoderm stem cell with self-replication and multiple differentiation potential, can differentiate into bone, cartilage, or fat cells [7, 8]. Therefore, stem cell transplantation and/or gene-enhanced cartilage tissue may become potential methods for cartilage repair [9].

Bone morphogenetic proteins (BMPs) are growth and differentiation factors that belong to the transforming growth factor-β superfamily [10]. Bone morphogenetic protein 2 (BMP2), one of about 30 distinct BMPs [11], plays an important role in inducing osteogenesis and chondrogenesis of stem cells [12–14]. BMP2 has been proven to induce chondrogenic differentiation of human synovial MSCs in a dose-dependent manner and to be more capable of inducing chondrogenic differentiation than many other growth factors, such as transforming growth factor-β and insulin-like growth factor-1 (IGF-1) [15].

Oxidative stress has been widely proven to contribute to degeneration and injury [16]. The reactive oxygen species and free radicals produced in the process of oxidative stress can cause oxidative stress damage and cell death [17]. Cartilage repair techniques must take into account the ongoing inflammatory microenvironment that occurs during the course of osteoarthritis and injury. Therefore, it is of great significance to explore the molecular mechanisms and synergistic effects involved in chondrogenic differentiation of stem cells and blocking the oxidative stress microenvironment in order to create a more suitable microenvironment for the chondrogenesis of MSCs during cartilage repair.

Silent mating type information regulator 2 homolog-1 (SIRT1), an NAD-dependent class III histone deacetylase, deacetylated histone and non-histone proteins play important roles in the coordination of cellular functions, such as cell differentiation, proliferation, aging, apoptosis, and oxidative stress [18–20]. Studies have reported that the protein levels and activity of SIRT1 are reduced significantly during the development of osteoarthritis [21, 22]. The expression of SIRT1 is also significantly reduced in cartilage endplates in intervertebral disc degeneration [19, 23]. SIRT1 has also been proven to modify Sox9 by deacetylation, which can promote the chondrogenic differentiation of stem cells [24, 25]. In addition, SIRT1 can affect deacetylation and nuclear translocation of nuclear factor-kappa B (NF-κB) subunit Rel/p65, thereby reducing the apoptosis rate and improving antioxidant activity of cells in inflammation and aging [26, 27]. However, the role of SIRT1 in chondrogenic differentiation and cartilage maintenance in MSCs is poorly understood. Thus, we conducted the present study to detect whether SIRT1 could coordinate BMP2-induced chondrogenic differentiation, reducing apoptosis and the decomposition of extracellular matrix under oxidative stress.

## RESULTS

### The C3H10T1/2 cells infected with Ad-BMP2, Ad-SIRT1, or Ad-GFP exogenously expressed BMP2 and SIRT1

**Figure 1:**
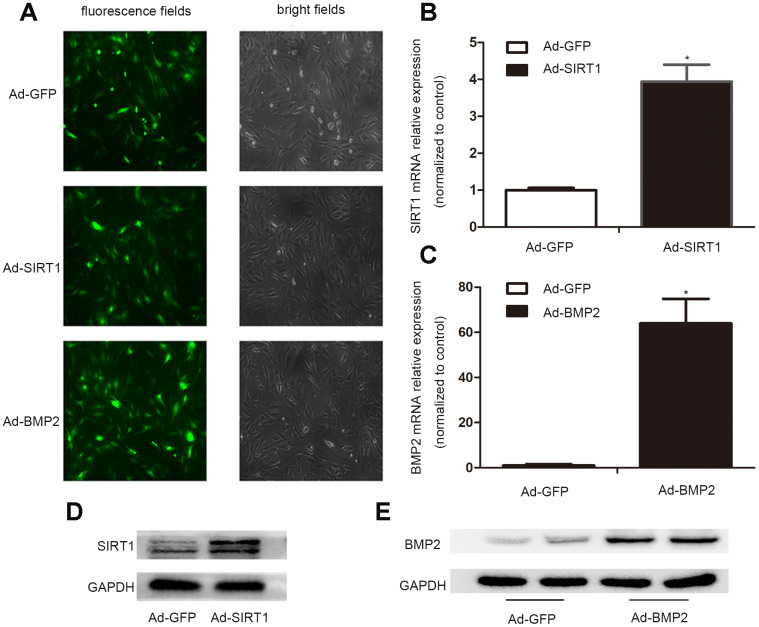
**The successful infection of the C3H10T1/2 cells with Ad-SIRT1, Ad-BMP2, and Ad-GFP.** (**A**) C3H10T1/2 cells were infected with Ad-SIRT1, Ad-BMP2, or Ad-GFP in plate cultures. Bright light and fluorescence microscopy (100X) examination showed the efficiency of recombinant adenoviruses after 24 h of virus infection. (**B**, **C**) Total RNA was isolated 24 h after infection, and the mRNA expression of SIRT1 and BMP2 was measured using real-time PCR. (**D**, **E**) The SIRT1 and BMP2 protein levels were measured after 48 h of infection using the western blotting method. The data are denoted as the mean ± SD.*: p < 0.05 vs. Ad-GFP.

In order to obtain high levels of transgene expression, we constructed recombinant adenoviruses expressing Ad-BMP2, Ad-SIRT1, and Ad-GFP. After 24 h of virus infection, the morphology of the C3H10T1/2 cells was observed under a fluorescence microscope, and the intensity of fluorescence infection showed that the C3H10T1/2 cells were successfully infected with adenovirus (Figure 1A). The results of real-time fluorescence quantitative PCR (Figure 1B, 1C) and western blotting showed that C3H10T1/2 cells transfected with the Ad-BMP2 and Ad-SIRT1 highly expressed BMP2 and SIRT1, but this did not occur in cells infected with Ad-GFP (Figure 1D, 1E). These data indicated that we successfully constructed recombinant adenoviruses of Ad-BMP2, Ad-SIRT1, and Ad-GFP and effectively infected the C3H10T1/2 cells.

### SIRT1 potentiated BMP2-induced chondrogenic differentiation of the C3H10T1/2 cells in vitro

**Figure 2:**
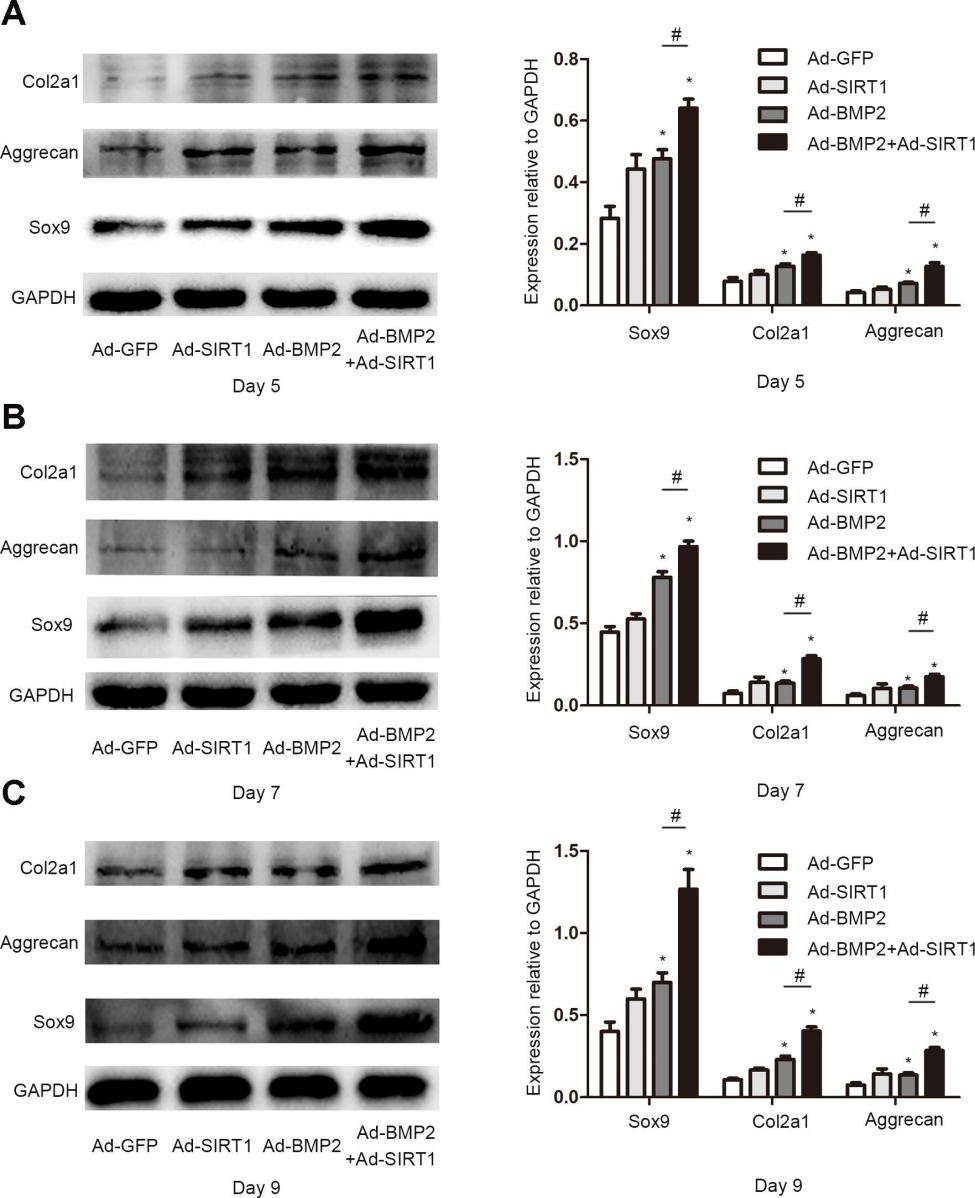
**SIRT1 potentiated the BMP2-induced expression of chondrogenic differentiation makers.** (**A**) The Sox9, Col2a1, and aggrecan protein levels in C3H10T1/2 cells infected with Ad-GFP, Ad-SIRT1, Ad-BMP2, or Ad-BMP2+Ad-SIRT1 for 5 days. (**B**) The Sox9, Col2a1, and aggrecan protein levels in C3H10T1/2 cells infected with Ad-GFP, Ad-SIRT1, Ad-BMP2, or Ad-BMP2+Ad-SIRT1 for 7 days. (**C**) The Sox9, Col2a1, and aggrecan protein levels in C3H10T1/2 cells infected with Ad-GFP, Ad-SIRT1, Ad-BMP2, or Ad-BMP2+Ad-SIRT1 for 9 days. All of the relative protein expression level results were compared to those of GAPDH using Quantity one. The data are denoted as the mean ± SD.*:p < 0.05 vs. Ad-GFP; #:p < 0.05, Ad-BMP2 group vs. Ad-BMP2+Ad-SIRT1 group.

**Figure 3:**
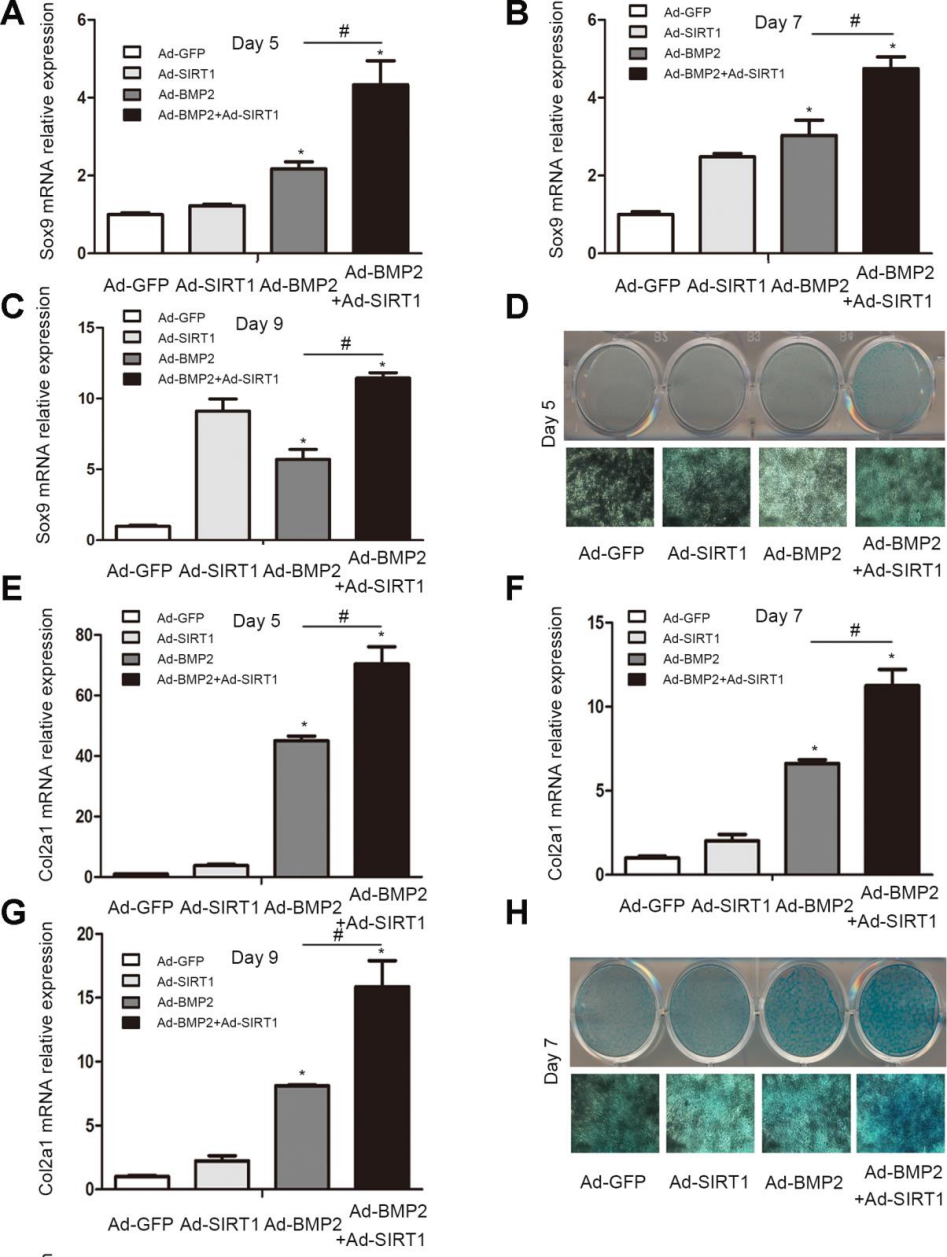
**The C3H10T1/2 cells were cultured in 6-well plates after being infected with Ad-GFP, Ad-SIRT1, Ad-BMP2, or Ad-BMP2+Ad-SIRT1.** (**A**–**C**) The Sox9 mRNA levels of C3H10T1/2 cells were measured after 5, 7 and 9 days using real-time PCR. (**E**–**G**) The Col2a1 mRNA levels of C3H10T1/2 cells were measured after 5, 7 and 9 days using real-time PCR. (**D**, **H**) Alcian blue staining was used to observe the secretion of extracellular matrix after the 5 and 7 days of Ad-GFP, Ad-SIRT1, Ad-BMP2, and Ad-BMP2+Ad-SIRT1 infection. The dyed 12-well plates were photographed by a camera attached to a light microscope (100X). All of the relative mRNA expression level results were compared to those of GAPDH using Quantity one. The data are denoted as the mean ± SD.*:p < 0.05 vs. Ad-GFP; #:p < 0.05, Ad-BMP2 vs. Ad-BMP2+Ad-SIRT1.

Sox9, a key regulator of early chondrogenic differentiation expression, was detected by western blotting on the 5^th^, 7^th^, and 9^th^ day after the C3H10T1/2 cells were infected with Ad-GFP, Ad-SIRT1, Ad-BMP2, or Ad-SIRT1+Ad-BMP2. We found that the expression of Sox9 in the Ad-SIRT1+Ad-BMP2 group was significantly higher than that in the Ad-BMP2 group, and the expression levels were significantly higher in both groups compared with that in the control group (Figure 2, P < 0.05). We also detected the Sox9 downstream regulatory genes type II collagen (Col2a1) and aggrecan using the western blotting method. The results showed that SIRT1 could significantly promote the expression of Col2a1 and aggrecan in BMP2-induced chondrogenic differentiation of the C3H10T1/2 cells (Figure 2, P < 0.05). At the same time, we examined the levels of Sox9 and Col2a1 mRNA on the 5^th^ (Figure 3A, 3E, p < 0.05), 7^th^ (Figure 3B, 3F, p < 0.05), and 9^th^ (Figure 3C, 3G, p < 0.05) day using real-time PCR. PCR analysis showed that the expression of cartilage markers (Sox9 and Col2a1) in both the Ad-SIRT1+Ad-BMP2 group and Ad-BMP2 group was significantly higher than that in the control group, and the expression of cartilage makers in the Ad-SIRT1+Ad-BMP2 group was significantly higher than that in the Ad-BMP2 group. In addition, Alcian blue staining was used to observe the secretion of extracellular matrix after the 5^th^ (Figure 3D) and 7^th^ (Figure 3H) day of adenovirus infection. The results showed that cells in the Ad-BMP2+Ad-SIRT1 group secreted more extracellular matrix than those in the BMP2 group.

### SIRT1 potentiated BMP2-chondrogenic differentiation of C3H10T1/2 cells in vivo

**Figure 4:**
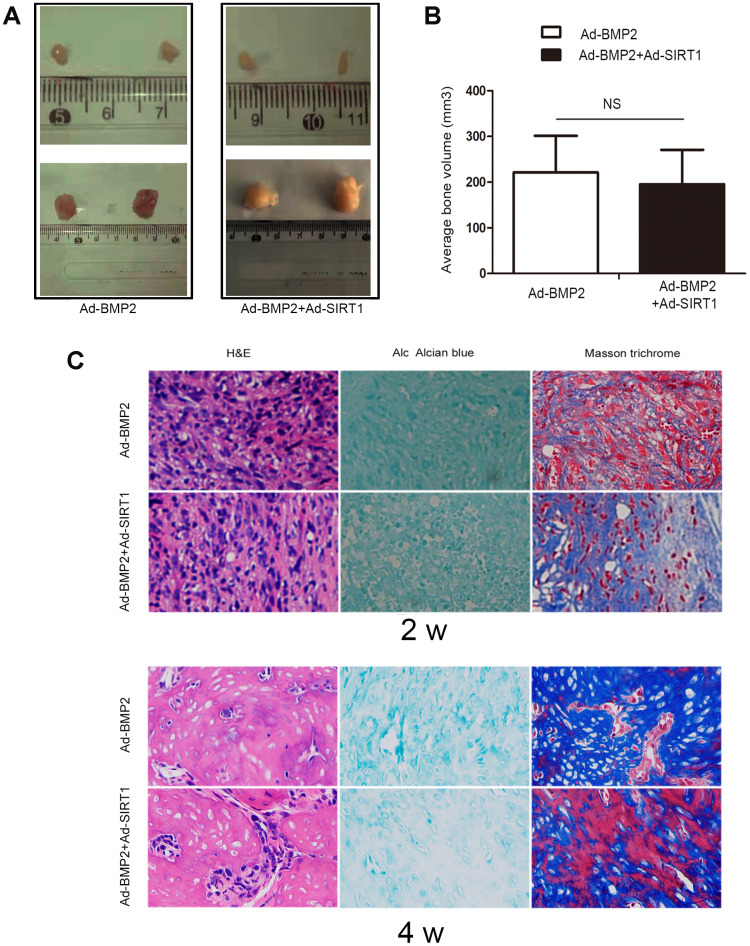
**SIRT1 potentiated BMP2-induced chondrogenesis in vivo.** (**A**) Macrographic images of the ectopic bone mass. The ectopic osseous masses were retrieved after 2 and 4 weeks. (**B**) The bone mass volumes were evaluated. (**C**) The bone masses retrieved from the Ad-BMP2 and Ad-BMP2+Ad-SIRT1 group were fixed and decalcified, and hematoxylin and eosin, Alcian blue, and Masson trichrome staining was performed on the extracted bony mass (200X). NS: p > 0.05.

In order to verify the effect of SIRT1 on BMP2-induced chondrogenic differentiation of stem cells, we also carried out stem cell transplantation experiments. After 2 and 4 weeks of injection with C3H10T1/2 cells, the animals were euthanized, and the bony mass was removed. The C3H10T1/2 cells infected with either Ad-SIRT1 or Ad-GFP alone did not yield any detectable masses (Figure 4A). We measured the bone tissue size of the Ad-BMP2 and Ad-BMP2+Ad-SIRT1 group, and there was no significant difference between them (Figure 4B). Hematoxylin and eosin, Alcian blue, and Masson trichrome staining were performed on the extracted bony mass (Figure 4C). The staining showed that the cartilage morphology including chondrocytes and chondrocyte lacunae in the Ad-SIRT1+Ad-BMP2 group was more obvious than that in the Ad-BMP2 group after 2 weeks of injection. At the fourth week, the cartilage lacuna and differentiation of bone tissue were more obvious compared in two weeks. Simultaneously, in the Ad-SIRT1+Ad-BMP2, the bone tissue was more mature and differentiated into osteoblasts compared with group infected with BMP2 in four weeks.

### SIRT1 alleviated the apoptosis of BMP2-induced chondrogenic differentiation stem cells under oxidative stress

**Figure 5:**
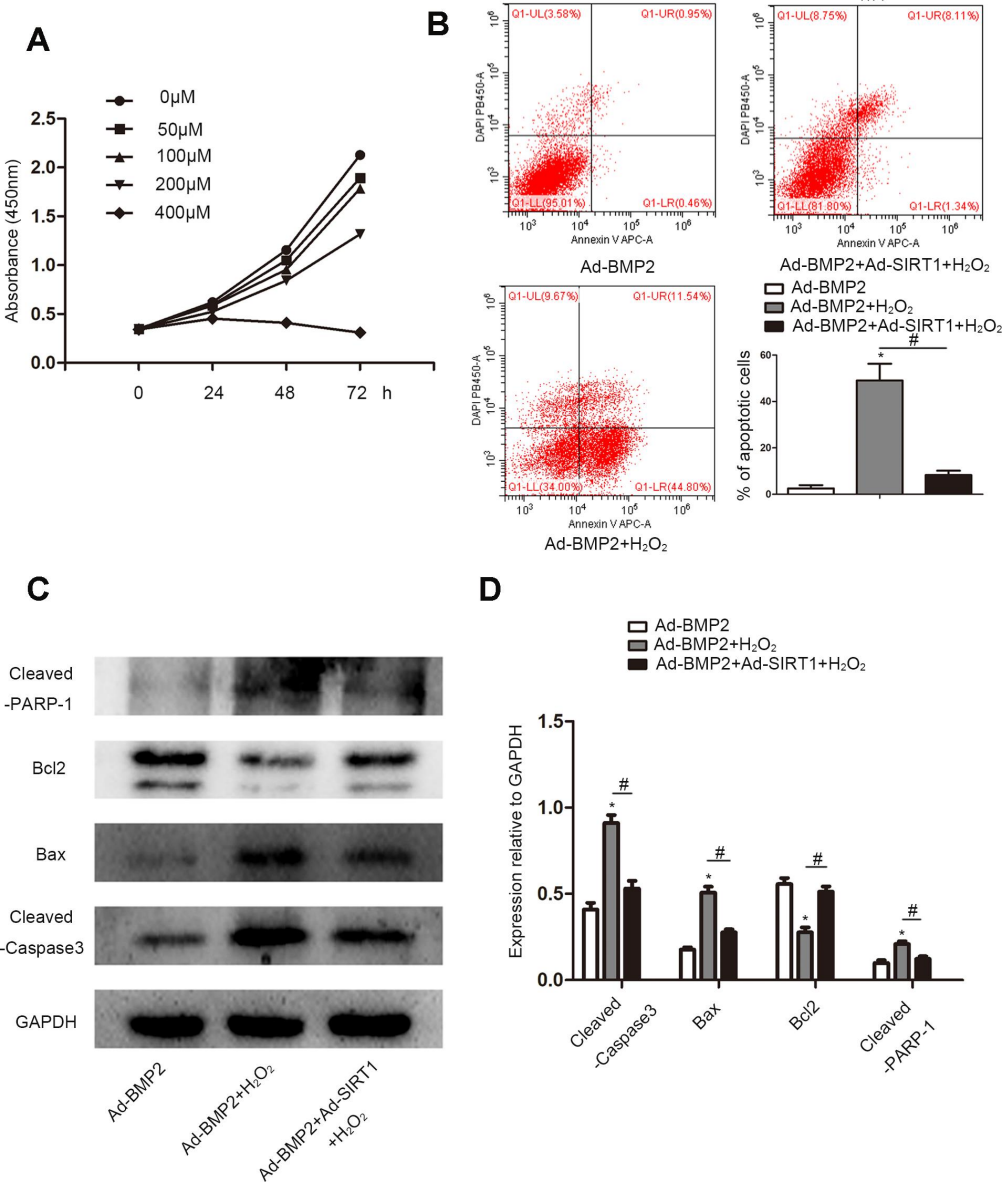
**SIRT1 alleviated the apoptosis of BMP2-induced chondrogenic differentiation stem cells under oxidative stress.** (**A**) Cell Counting Kit-8 assay to detect cell proliferation under different concentrations (0, 50, 100, 200, or 400 μM) of H_2_O_2_. (**B**) The cell apoptotic rates were measured using a flow cytometry assay under 200 μM of H_2_O_2_. (**C**, **D**) H_2_O_2_ increased the expression of Cleaved-Caspase3, Bax, and Cleaved-PARP-1 and reduce the expression of Bcl-2 compare to the control group. The Ad-BMP2+Ad-SIRT1+H_2_O_2_ group exhibited opposite results. The data are denoted as the mean ± SD.*: p < 0.05 Ad-BMP2+H_2_O_2_ vs. Ad-BMP2; #: p < 0.05, Ad-BMP2 + H_2_O_2_ vs. Ad-BMP2+Ad-SIRT1+H_2_O_2_.

CCK8 was used to detect apoptotic cells treated with different concentrations (0, 50, 100, 200, and 400 μM) of H_2_O_2_ (Figure 5A). With the increase in H_2_O_2_ concentration, the decrease of cell absorbance at 450 nm indicated an increase in cell apoptosis. We selected the appropriate concentration (200 μM) according to the results of the CCK8 assay for subsequent experiments. We used flow cytometry to detect apoptosis of Ad-BMP2, Ad-BMP2+H_2_O_2_, and Ad-BMP2+Ad-SIRT1+H_2_O_2_ cells (Figure 5B). The results showed that H_2_O_2_ could promote cell apoptosis, but the apoptotic rate of the Ad-BMP2+Ad-SIRT1+H_2_O_2_ group was significantly lower than that of the Ad-BMP2+H_2_O_2_ group. In addition, we detected apoptosis-related proteins (Bcl-2, Bax, Cleaved-PARP-1, Cleaved-Caspase3) using the western blotting method (Figure 5C, 5D). The results showed that the expression of Bax, Cleaved-PARP-1, and Cleaved-Caspase3 proteins increased and the expression of Bcl-2 protein decreased in the Ad-BMP2+H_2_O_2_ group compared with the Ad-BMP2 group, and the Ad-BMP2+Ad-SIRT1+H_2_O_2_ group exhibited opposite results.

### SIRT1 inhibited the decomposition of extracellular matrix in BMP2-induced-C3H10T1/2 cells under oxidative stress

**Figure 6:**
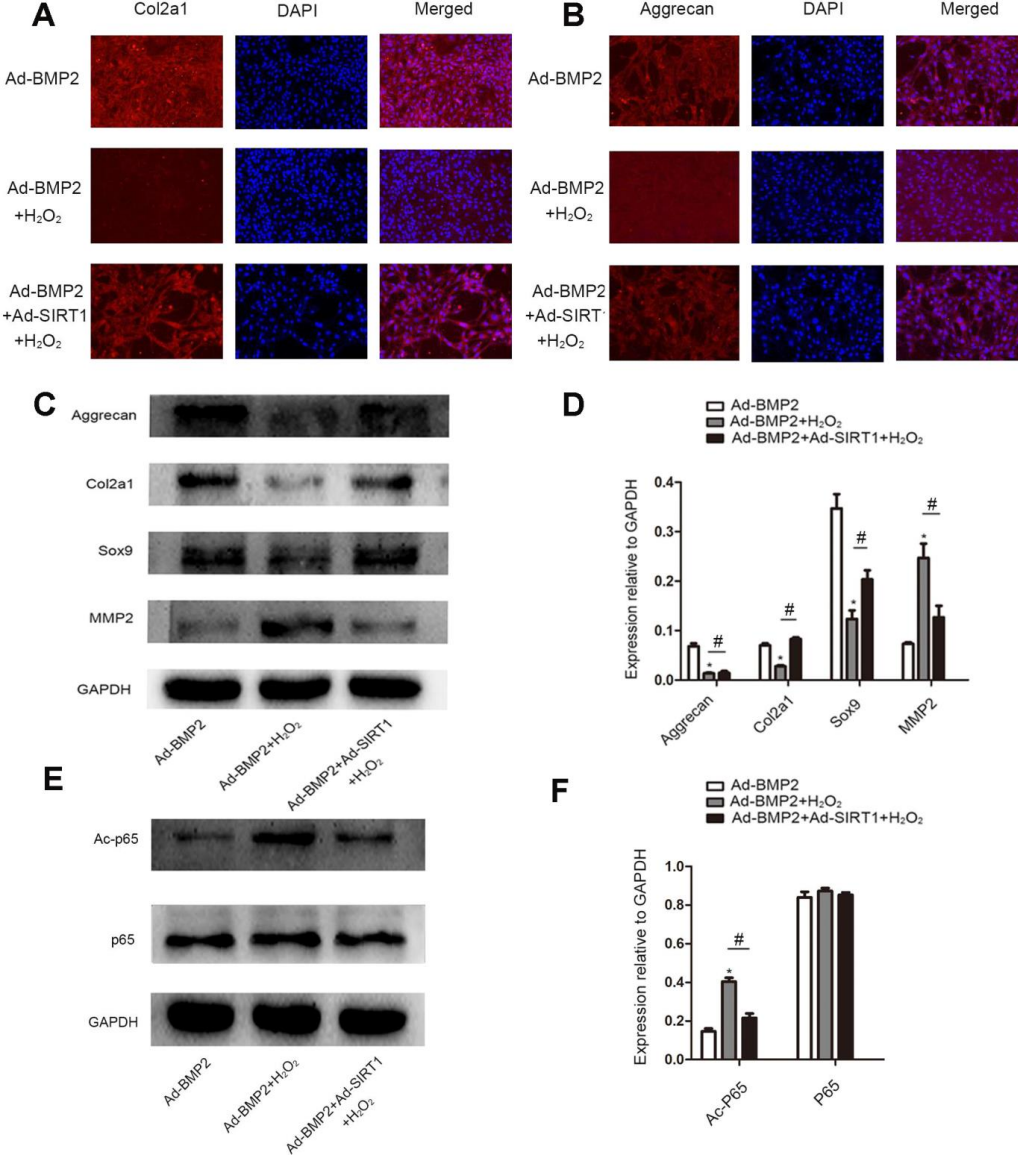
**SIRT1 inhibited the decomposition of extracellular matrix in BMP2-induced-C3H10T1/2 cells under oxidative stress.** (**A**, **B**) Immunofluorescence analysis of the expression of Col2a1 and aggrecan among the Ad-BMP2, Ad-BMP2+H_2_O_2_, and Ad-BMP2+Ad-SIRT1+H_2_O_2_ groups (200X). The Col2a1 and aggrecan proteins were labeled in red, and the nuclei were labeled in blue by DAPI staining. (**C**, **D**) The expression of Sox9, Col2a1, and aggrecan proteins decreased in the Ad-BMP2+H_2_O_2_ group compared with the Ad-BMP2 group, while the expression of Sox9, Col2a1, and aggrecan proteins increased in the Ad-SIRT1+Ad-BMP2+H_2_O_2_ group compared with that of the Ad-BMP2+H_2_O_2_ group. The expression of MMP2 increased in the Ad-BMP2+H_2_O_2_ group compared with that in the Ad-BMP2 group, while the expression of MMP2 in the SIRT1+BMP2+H_2_O_2_ group was significantly lower than that of the Ad-BMP2 group. (**E**, **F**) The expression of Ac-p65 and p65 among Ad-BMP2, Ad-BMP2+H_2_O_2_, and Ad-BMP2+Ad-SIRT1+H_2_O_2_ groups. The data are denoted as the mean ± SD. *: p < 0.05, Ad-BMP2+H_2_O_2_ vs. Ad-BMP2; #: p < 0.05, Ad-BMP2+Ad-SIRT1+H_2_O_2_ vs. Ad-BMP2+H_2_O_2_.

We detected the secretion of extracellular matrix (Col2a1, aggrecan) in BMP2-induced C3H10T1/2 cells treated with 200 μM of H_2_O_2_ by an immunofluorescence assay (Figure 6A, 6B). The results showed that there were less Col2a1 and aggrecan proteins in the Ad-BMP2+H_2_O_2_ group than in the Ad-BMP2 group, while there were more proteins expressed in the Ad-SIRT1+Ad-BMP2+H_2_O_2_ group than in the Ad-BMP2+H_2_O_2_ group. At the same time, we detected the synthesis and decomposition of extracellular matrix proteins (Sox9, Col2a1, aggrecan, MMP2) by the western blotting method (Figure 6C, 6D). The results showed that the expression of Sox9, Col2a1, and aggrecan proteins decreased and the expression of MMP2 increased in the Ad-BMP2+H_2_O_2_ group compared with the Ad-BMP2 group, and Ad-BMP2+Ad-SIRT1+H_2_O_2_ group showed opposite results. We also detected the acetylation level of p65 (Figure 6E, 6F) and found that the acetylation level of p65 increased after adding H_2_O_2_ stimulation, while the acetylation level of p65 in the Ad-SIRT1+BMP2+H_2_O_2_ group was significantly lower than that of the Ad-BMP2+H_2_O_2_ group. These results suggested that SIRT1 not only alleviated apoptosis but also inhibited the decomposition of extracellular matrix in BMP2-induced chondrogenesis in C3H10T1/2 cells under oxidative stress.

## DISCUSSION

Cartilage injury is a common joint disease in the clinic, and cartilage tissue engineering provides a new treatment option for cartilage injury repair [30]. MSCs have been used as seed cells because of their wide range of sources, easiness to obtain and expand *in vitro*, and ability to be used for autotransplantion because of their low immunogenicity [7, 8]. MSCs can differentiate into chondrocytes under certain conditions, which could provide numerous raw materials for the treatment of cartilage injury diseases, and tissue engineering promotes joint repair by introducing seed cells, biomaterials, and growth factors into damaged joint tissues [8, 31].

BMP2, a member of the TGF-β super-family, plays an essential role in the regulation of chondrocyte proliferation and maturation during endochondral bone development [32]. It has been proven to be more capable of inducing osteochondrogenic development and MSC chondrogenic differentiation than other growth factors, such as BMP4, BMP6, BMP7, TGF-βs, and IGF-1 [15, 33]. In our previously study [12] and this study, we confirmed that BMP2 can up-regulate Sox9 expression, which is a key regulator of chondrogenic differentiation of stem cells, and also increase the expression of downstream genes including Col2a1 and aggrecan.

However, inflammation and oxidative stress significantly inhibit chondrogenic differentiation of stem cells, and NF-κB is the key transcription factor that mediates inflammatory and oxidative stress responses [20]. IL-1β, TNF-α, and oxidative stress can also directly inhibit stem cell chondrogenic differentiation through the NF-κB signaling pathway [34, 35]. Inflammation and oxidative stress are involved in tissue degeneration and injury [16]. Cartilage repair approaches must address the ongoing inflammatory microenvironment that occurs during the course of osteoarthritis and injury. So, it is of great significance to explore the molecular mechanism and synergistic effects of chondrogenic differentiation of stem cells and blocking the oxidative stress microenvironment in order to create a more suitable microenvironment for the chondrogenesis of MSCs during cartilage repair. In this study, we explored the effect of SIRT1 on BMP2-induced chondrogenic differentiation of stem cells and the effect of SIRT1 on the homeostasis of BMP2-induced chondrogenic differentiation of stem cells in the oxidative stress microenvironment.

We found that SIRT1 could significantly increase the expression of Sox9, Col2a1, and aggrecan in BMP2-induced stem cell differentiation. This is in accordance with the result that SIRT1 can deacetylate Sox9 and enhance transcriptional activity of Sox9, promoting chondrogenic differentiation of stem cells [24]. We also confirmed that SIRT1 can significantly promote BMP2-induced chondrogenic differentiation of stem cells and the formation of the cartilage structure through stem cell transplantation experiments *in vivo*. The reactive oxygen species in oxidative stress can cause oxidative stress damage and cell death. We found that SIRT1 could significantly decrease the apoptosis rate during BMP2-induced chondrogenic differentiation under oxidative stress. In addition, SIRT1 could significantly decrease the expression extracellular matrix enzymes (MMP2) and reverse the decrease of Sox9, Col2a1, and aggrecan expression in oxidative stress. Therefore, SIRT1 may be an important synergistic factor in BMP2-induced chondrogenesis of stem cells and plays an important role in blocking the oxidative stress microenvironment to create a more suitable microenvironment for the chondrogenesis of MSCs.

Protein deacetylation modification regulates multiple functions [36], and there are few studies regarding its role in stem cell chondrogenic differentiation. Our study innovatively explored the regulatory role of deacetylation-modifying enzyme SIRT1 in BMP2-induced chondrogenic differentiation of stem cells, which may help to further reveal the mechanism of BMP2-induced chondrogenic differentiation of stem cells, and the new synergistic factors for BMP2-induced stem cell chondrogenic differentiation. Moreover, an oxidative stress environment is an inhibitory factor in cartilage repair. Therefore, this study innovatively explored the role of SIRT1 in the maintenance of cartilage homeostasis in BMP2-induced stem cell chondrogenic differentiation under oxidative stress, which provided new methods to overcome the negative effects of oxidative stress in cartilage repair.

In conclusion, our study revealed that SIRT1 can promote BMP2-induced chondrogenic differentiation of stem cells and reduce the cell apoptosis and the decomposition of extracellular matrix due to NF-κB/p65 deacetylation under oxidative stress, maintaining the cartilage phenotype. SIRT1 may be a new candidate to promote chondrogenesis and overcome the negative effects of oxidative stress during cartilage repair.

## MATERIALS AND METHODS

### HEK-293 cells and the C3H10T1/2 cell line culture

The HEK-293 cells and mesenchymal stem cell line (C3H10T1/2) used in this study were purchased from the American Type Culture Collection (ATCC; USA). They were maintained in Dulbecco’s modified Eagle’s medium (DMEM, Hyclone, USA) supplemented with 10% fetal bovine serum (FBS, Biological Industries, Israel), 100 U/mL streptomycin, and 1% penicillin (Beyotime, China). The cells were incubated in an atmosphere containing 5% CO_2_ at 37°C.

### Recombinant adenoviruses expressing GFP, BMP2, and SIRT1 and cell transfection

The human SIRT1 was polymerase-chain-reaction (PCR)-amplified, cloned into an adenoviral shuttle vector, and subsequently used to generate recombinant adenoviruses in the HEK-293 cells. The primers used to amplify the coding regions included SIRT1 F:5’-ACAATTTAAATATGATTGGCACAGATCCTC-3’and R:5’-ATTAGCGGCCGCTGAT TTGTTTGATGGA-3’. SwaI and NotI enzymes were used to clone the BMP2 coding regions into the shuttle vector. The resulting adenoviruses were denoted as Ad-SIRT1 and Ad-GFP. The green fluorescent protein (GFP) was used to monitor infection efficiency via fluorescence microscopy (Nikon TE200-U, Japan). Ad-SIRT1, which expressed the SIRT1 protein, also expressed GFP. Ad-GFP, which only expressed GFP, was used as the control. The Ad-BMP2 was denoted by TC He, and it has been used in many studies [12, 14, 28].

### Alcian blue staining

The C3H10T1/2 cells were seeded in a 12-well plate; infected with Ad-GFP, Ad-SIRT1, Ad-BMP2, or Ad-BMP2+Ad-SIRT1; and subsequently were stained with Alcian blue on the 5^th^ and 7^th^ day to detect the secretion of extracellular matrix. The plates were washed with phosphate buffered saline (PBS), fixed in 4% paraformaldehyde for 30 min, and washed again with PBS. The cells were stained with Alcian blue solution (Solarbio, China) according to the instructions and then photographed.

### Cell proliferation assay

The cell counting kit-8 assay (CCK8, MedChemexpress, USA) was used to investigate cell proliferation. The C3H10T1/2 cells were seeded in 96-well plates at a density of 2000 cells/well, following treatment with H_2_O_2_ (0, 50, 100, 200, 400 μM). CCK8 reagent was added into wells, and cell proliferation was detected at 0, 24, 48, and 72 h by measuring the absorbance at 450 nm with a microplate reader.

### Apoptosis assay

Flow cytometry was used to detect cell apoptosis. Cells were processed in 6-well plates for 48 h. Cells were digested with 0.25% EDTA-free trypsin, washed twice with PBS, and collected in 1.5 ml EP tubes. Annexin V APC-A (Threebond, China)/DAPI (Beyotime, China) was added according to the instructions, and the apoptosis was examined by a flow cytometer. The stained cells were subjected to flow cytometry to identify live cells (Annexin V APC-A-/DAPI PB 450-A-), early apoptotic cells (Annexin V APC-A+/DAPI PB 450-A-), late apoptotic or secondary apoptotic cells (Annexin V APC-A+/DAPI PB 450-A+), and necrotic cells (Annexin V APC-A-/DAPI PB 450-A+).

### Immunofluorescence assay

The C3H10T1/2 cells were seeded in a 6-well plate with glass coverslips and infected with Ad-BMP2, Ad-BMP2+H_2_O_2_, or Ad-BMP2+Ad-SIRT1+H_2_O_2_ for 3 days. The cells were washed with PBS, fixed with 4% paraformaldehyde for 30 min at room temperature, and again washed with PBS. The cells were permeabilized with 0.1% Triton X-100 (Beyotime, China) at room temperature for 20 min, blocked with 1% bovine serum albumin (Beyotime, China) for 1 h, and incubated with primary antibody (anti-Col2a1, 1:200; anti-Aggrecan, 1:50) at 4°C overnight. Subsequently, the cells were incubated with Alexa Fluor®594-conjugated secondary antibody for 1 h in the dark at 37°C and washed with PBS. Finally, the nuclei were stained with DAPI (Beyotime, China) for 5 min in the dark.

### Subcutaneous stem cell implantation

The C3H10T1/2 cells were infected with Ad-GFP, Ad-SIRT1, Ad-BMP2, or Ad-BMP2+Ad-SIRT1 at a density of 70%. Twenty four hours after infection, the cells were digested, collected, and resuspended with PBS to 5 × 10^7^ cells/ml. Each athymic nude mouse (groups of 4 to 6 week-old males, Beijing HFK Bioscience Corporation, China) was injected with 100 μl PBS (about 5 × 10^6^ cells), subcutaneously. After two or four weeks of subcutaneous implantation, nude mice were euthanized, and bone mass was removed. All of the experiments involving animals were performed according to institutional animal guidelines (Ethics Committee of Chongqing Medical University).

### Hematoxylin and eosin, alcian blue, and Masson trichrome staining

The retrieved tissues were fixed in 4% paraformaldehyde, decalcified, and embedded in paraffin. Hematoxylin and eosin, Alcian blue, and Masson’s trichrome staining were performed as described [29].

### Real-time PCR

Total RNA was extracted with TRIzol® reagent (Takara, China), following the manufacturer's protocols. Then, the total RNA was reverse transcribed into cDNA using the PrimeScript™ RT reagent kit (Takara, China). Finally, the cDNA was amplified using SYBR Green Super Mixture, and PCR was conducted with a CFX-Connect Real-Time PCR system (Bio-Rad). The amplification conditions were as follows: 95°C for 30 sec, followed by 40 cycles of 95°C for 5 sec and 60°C for 1 min, and the melt curve at 60°C to 95°C, with an increment of 0.5°C for 5 sec. The primer sequences were as follows:

Sirt1-F: 5’-TGATTGGCACCGATCCTCG-3’, Sirt1-R: 5’-CCACAGCGTCATATCATCCAG-3’; BMP2-F: 5’-GGGACCCGCTGTCTTCTAGT-3’, BMP2-R: 5’-TCAACTCAAATTCGCTGAGGAC-3’; Col2A1-F: 5’-GGGTCACAGAGGTTACCCAG-3’, Col2A1-R: 5’-ACCAGGGGAACCACTCTCAC-3’; Sox9-F: 5’-AGTACCCGCATCTGCACAAC-3’, Sox9-R: 5’-ACGAAGGGTCTCTTCTCGCT-3’; GAPDH-F: 5’-AGGTCGGTGTGAACGGATTTG-3’, GAPDH-R: 5’-GGGGTCGTTGATGGCAACA-3’.

### Western blotting analysis

Proteins were extracted with lysis buffer and phenylmethanesulfonyl fluoride (Beyotime, China). A bicinchoninic acid kit (Beyotime, China) was used to detect the protein concentration. Total cell proteins were separated by SDS-PAGE (Beyotime, China) and transferred to a polyvinylidene fluoride membrane. The membranes were blocked in 5% skimmed milk for 1 h at room temperature and incubated in specific antibodies at 4°C overnight. The blots were washed three times with TBST and incubated in secondary antibodies for 1 h at room temperature. The Immobilon Western Chemiluminescent Kit (NCM Biotech, China) was used to detect protein levels. The primary antibodies used included: anti-aggrecan (Santa Cruz Biotechnology, USA; 1:500), anti-Sox9 (Abcam, USA; 1:1000), anti-Bcl-2 (Abcam, USA; 1:2000), anti-Bax (Abcam, USA; 1:2000), anti-SIRT1 (Abcam, USA; 1:1000), anti-GAPDH (Abcam, USA;1:10000), anti-NF-κB p65 (Abcam, USA; 1:1000), anti-Acetyl-NF-κBp65 (Lys310) (Affinity Biosciences, USA; 1:500), anti-BMP2 (Immunoway, USA; 1:500), anti-Col2a1 (Immunoway, USA; 1:500), anti-MMP2 (Immunoway, USA; 1:500), anti-Cleaved-Caspase3 (Immunoway, USA; 1:1000), and anti-Cleaved-PARP-1 (Immunoway, USA; 1:500).

### Statistical analysis

Experiments were repeated three times, and the data are expressed as the mean ± standard deviation (SD). Any statistically significant differences between groups were evaluated using a one-way variance analysis (SPSS cooperation, SPSS 17.0). P < 0.05 was considered to indicate a statistically significant difference.
